# Effects of mixed selenium sources on the physiological responses and blood profiles of lactating sows and tissue concentration of their progeny

**DOI:** 10.5713/ab.22.0106

**Published:** 2022-06-30

**Authors:** Cheon Soo Kim, Xing Hao Jin, Yoo Yong Kim

**Affiliations:** 1 Department of Agricultural Biotechnology and Research Institute of Agriculture and Life Science, Seoul National University, Seoul 08826, Korea

**Keywords:** Piglets, Selenium, Serum, Sow, Sow Milk, Tissues

## Abstract

**Objective:**

This study was conducted to investigate the effects of selenium benefits on the physiological responses, litter performance, blood profiles and milk composition of lactating sows and tissue concentration of their progeny when mixed form of selenium was provided in a lactation diet.

**Methods:**

A total of 45 multiparous sows (Yorkshire×Landrace) with similar body weight, backfat thickness, and parity were assigned to one of three treatments with 15 sows per treatment in a completely randomized design. Organic and inorganic selenium were mixed and added to the diet at 0.15 ppm and 0.25 ppm, respectively. A non-Se-fortified corn–soybean meal basal diet served as a negative control. Treatments were as follows: i) Control: corn–soybean meal based diet, ii) ISOS15: control+ inorganic Se 0.15 ppm+organic Se 0.15 ppm, iii) ISOS25: control+inorganic Se 0.25 ppm+organic Se 0.25 ppm.

**Results:**

Serum selenium concentrations of sows and piglets were increased by the supplemental Se mixture at 7 days of lactation compared with the control (p<0.01, respectively). The kidney and muscle selenium concentrations of piglets were increased by the supplemental Se mixture at 21 days of lactation compared with the control (p = 0.03; p = 0.04, respectively).

**Conclusion:**

Consequently, supplementation with mixed inorganic and organic selenium in a lactating diet could improve the selenium status of sows and piglets; no differences were observed among the mixing levels.

## INTRODUCTION

As consumers’ interest in well-being has increased recently and many foods with enhanced physiologically active substances are on the market according to this trend. In particular, in livestock foods, research is actively being conducted to produce foods with fortified physiologically active substances. In Korea, omega eggs [1], conjugated linoleic acid-reinforced pork and eggs [2,3], and selenium-reinforced eggs and pork are being produced, and interest in trace nutrients is increasing overseas [4].

Among the functional substances added to livestock feed, selenium (Se) is under the spotlight, as well as vitamin C and E, which have been proven to have antioxidant ability in the animal body. When livestock are supplied with selenium sources (organic and inorganic), it is reported that organic selenium has a higher retention rate and accumulation efficiency in the intestines than inorganic selenium [5,6]. As a result, organic selenium is used to produce selenium-rich livestock products, but organic selenium is not only highly dependent on imports, but also has a low added amount, which causes toxicity risks and expensive problems when consumed in excess [7]. Conversely, inorganic selenium is simple and inexpensive, but highly toxic. In addition, the amount of inorganic Se in the diet is difficult to determine and the absorption and conversion rates are low [8]. However, information on the effects of the mixed addition of organic and inorganic selenium on sows and their offspring is very limited.

The objective of this study was to investigate the effects of selenium supplementation on lactating sows, considering the physiological responses, litter performance, blood profiles and milk composition of lactating sows and tissue concentration of their progeny when a mixed form of selenium was provided in a lactation diet.

## MATERIALS AND METHODS

### Animals

All experimental procedures involving animals were conducted following the Animal Experimental Guidelines provided by the Seoul National University Institutional Animal Care and Use Committee (SNUIACUC; SNU-201112-3).

A total of 45 F1 multiparous sows (Yorkshire×Landrace) with an average body weight (BW) of 241.8 kg, average backfat thickness of 18.9 mm, and average parity of 3.5 were allotted to one of three treatments in a completely randomized design. All sows took two artificial insemination services according to the estrus cycle after weaning and had their pregnancy checked at day 35 of gestation using an ultrasound scanner (Dongjin BLS, Gwangju-si, Gyeonggi-do, Korea). Before starting the experiment, sows of second parity were fed a 2.2 kg/d gestation diet, and sows of over third parity were fed a 2.4 kg/d gestation diet.

### Experimental design and diet

All experimental diets for lactating sows were formulated based on corn–soybean meal and selenium was supplemented by source and level. Inorganic (sodium selenite) or organic (Se-enriched yeast) selenium sources were mixed and added to the diet at 0.15 or 0.25 ppm, respectively. A non-Se premix corn–soybean meal basal diet served as a negative control. The experimental selenium sources added to the diets were sodium selenite (1,000 ppm, inorganic Se) and selenium yeast (1,000 ppm, organic Se, Sel-Plex; Alltech, Lexington, KY, USA). The analyzed value of selenium in the basal diet was 0.071 ppm Se. All experimental lactation diets were formulated to contain 3,300 kcal of metabolizable energy ME/kg, 13.43% crude protein, 0.96% lysine, 0.26% methionine, 0.76% calcium and 0.65% total phosphorus. All other nutrients were formulated to meet or exceed the NRC [9] requirements. Chemical compositions of the experimental diet for lactating sows are presented in (Table 1).

### Animal management

All experimental sows (parity: 3 to 6) were fed the same commercial diet once a day at 08:00 h, providing 2.4 kg per day during gestation and decreasing gradually to 0.2 kg per day for 5 days before farrowing. After farrowing, sows were fed an experimental diet at the level of 1 to 5 kg/d for 5 days postpartum and fed diet *ad libitum* thereafter until weaning.

All sows were accommodated in individual gestation stalls (2.20×0.64 m), where the indoor temperature was regulated to an average of 20°C by an automatic ventilation system. At day 110 of gestation, sows were moved from the gestation barn to farrowing crates (2.50×1.80 m) after washing and disinfecting their body, especially the breast and vulva. None of the sows were treated with delivery inducer, and they were assisted when dystocia occurred. The room temperature of the lactating barn was kept at 28°C±2°C, and the baby house under a heating lamp was kept at 32°C±2°C. The air condition of the lactating barn was regulated automatically by the ventilation system and air conditioner.

After farrowing, piglets were cross-fostered until 24 h postpartum to balance the suckling intensity of the sows with equalization of the litter size to minimize any effect of initial litter size that could potentially affect litter growth. Cutting of the umbilical cord and tail and castration were conducted 3 days after birth, and piglets were injected with 150 ppm Fe-dextran (Gleptosil; Alstoe, York, UK). None of the piglets were fed creep feed during the whole lactation period. Weaning was performed at approximately 28 d.

### Body weight, backfat thickness, and lactation feed intake

The body weight and backfat thickness of the sows were measured at 24 h postpartum, and on 7 and 21 days of lactation. The body weight of the sow was measured by an electric scale (CAS Co. Ltd., Yangju-si, Gyeonggi-do, Korea) and backfat thickness was measured at the P2 position (mean value from both sides of the last rib and 65 mm away from the backbone) by an ultrasound device (Lean Meter; Renco Corp., Minneapolis, MN, USA). Daily feed wastage was recorded during lactation and lactation feed intake was measured when measuring the body weight and backfat thickness of lactating sows at day 21 of lactation.

### Litter performance

After farrowing, the number of piglets was recorded, and the body weight of live piglets was measured by an electric scale (CAS Co. Ltd., Korea). When measuring the body weight of piglets, ear notching was practiced for the experiment. Then, cross-fostering of the piglets was performed among all piglets until 12 h postpartum to equalize litter size. The number and body weight of the piglets were measured again on 7 and 21 days of lactation to calculate the litter weight, piglet weight, and both weight gain.

### Milk composition

Colostrum and milk samples were taken from functional mammary glands of each sow at 24 h postpartum, and on 7 and 21 days of lactation, respectively. Five milliliters of oxytocin (Komi oxytocin inj.; Komipharm International Co., Ltd., Siheung-si, Gyeonggi-do, Korea) was injected into the blood vessels of the sow’s ear.

Colostrum and milk were collected in 50 mL conical tubes (SPL Life Sciences Co., Ltd., Pocheon-si, Gyeonggi-do, Korea) from the first and second teats. After collection, samples were stored in a freezer (−20°C) until further analysis. Proximate analysis of colostrum and milk was conducted using Milkoscan FT120 (FOSS Electric, Hillerød, Denmark).

### Blood samples

Blood of sows (n = 4 for each treatment) was taken by venipuncture of the jugular vein using 10 mL disposable syringes at 24 h postpartum, and on 7 and 21 days of lactation. Blood of suckling piglets (n = 4 for each treatment) was collected from the anterior vena cava using 3 mL disposable syringes at 24 h postpartum and 5 mL disposable syringes on 7 days and 21 days of lactation.

All blood samples were enclosed in serum tubes (SST II Advance, BD Vacutainer; Becton Dickinson, Plymouth, UK) as well as ethylenediamine tetra-acetic acid tubes (BD Vacutainer K2E; Becton Dickinson, UK) and centrifuged at 3,000 rpm and 4°C for 15 min (5810R; Eppendorf, Hamburg, Germany) after clotting at room temperature for 30 min. The upper liquid (serum) of the blood was separated into a microtube (Axygen, Union City, CA, USA) and stored at −20°C until later analysis.

### Tissue samples

Piglets were killed and samples from the liver, kidney, and muscle were taken at 24 h postpartum (n = 4), and on 7 and 21 days of lactation (n = 3 for each treatment). Muscle samples were taken from the area of the muscle located between 5th and 6th ribs. Piglets were humanely euthanized through CO2 inhalation and exsanguination. Individual samples were stored at −20°C until further analysis.

### Se analytical methods

Colostrum, milk, serum and tissues were analyzed for Se using two methods, AOAC [10] and a microwave digestion method for selenium, which utilizes the microwave digestion with nitric acid and hydrogen peroxide. The ramp time was 15 min, the hold time was 20 min and the cool down time was 15 min with a temperature of 200°C. The samples were diluted into the linear calibration range with 1% methanol in water and analyzed on an ICP-MS (inductively coupled plasma source mass spectrometer). The canonical plotting standard from the ICP-MS was normalized to a rhodium internal standard. The ICP-MS instrument was Agilent 7500 (Santa Clara, CA, USA). Analyses of serum and milk samples from sows and pigs were conducted at the same time.

### Statistical analysis

All of the collected data were subjected to least squares mean comparisons and were evaluated with the general linear model (GLM) procedure of SAS (SAS Institute Inc., Cary, NC, USA). Individual sows, the whole litter weight, and the average pig weight within a litter were considered the experimental units. All collected data were analyzed by one-way analysis of variance. Contrasts were used to compare the control to other treatments. Differences among means were declared significant at p<0.05 and highly significant at p<0.01 and the determination of tendency for all analyses was p≥0.05 and p<0.10.

## RESULTS AND DISCUSSION

### Body weight, backfat thickness, and lactation feed intake of sows

The effects of dietary selenium in the lactation diet on the body weight and backfat thickness of sows during lactation are shown in (Table 2). The body weight, backfat thickness and lactation feed intake of sows were not affected by mixed addition levels of Se during lactation.

Previous studies, that examined effects of the addition of organic and inorganic Se mixture, reported that the sources and levels of Se did not affect the body weight, backfat thickness or average daily feed intake (ADFI) of lactating sows [11,12]. In agreement with the above, the present study indicates that the dietary Se source and level did not influence the body weight, backfat thickness, and lactation feed intake of sows during the whole experimental period.

In this study, when organic and inorganic Se were mixed and added to the lactating sow diet, the ISOS15 and ISOS25 treatments resulted in numerically higher ADFI than the control. The reason for the higher ADFI observed in ISOS15 and ISOS25 compared with the control remains uncertain. This could be related to the smell of the feed. The olfactory system of pigs is a fairly large, highly organized structure and is highly developed [13,14]. As smell is an initial attractant to feed, sows fed organic and inorganic Se may have consumed more feed due to attractive smell perception. Falk et al [15] observed higher feed intake in sows fed diets supplemented with SeMet (seleno-methionine). Nutrients supplied as feed are insufficient to maintain the sow's body shape and produce milk [16]. Decreased feed intake during the lactation period can lead to excessive weight loss and problems related to the reproduction of the next parity, resulting in delayed weaning-to-estrus interval [17], no estrus and a decrease in fertility [18]. Therefore, Eissen et al [19] reported that the feed intake of sows during lactation should be increased as much as possible for continuous reproduction.

### Litter performance

The effects of dietary selenium in the lactation diet on the performance of sows during lactation are shown in (Table 3). The number of piglets, litter weight, litter weight gain, average BW of piglets, and average BW gain of piglets were not affected by mixed addition levels of Se during lactation.

Organic Se supplementation during lactation has the potential to improve the growth performance of offspring [15, 20]. Additionally, Falk et al [21] reported that the growth performance of piglets was positively influenced by both the dietary Se source and level. However, previous studies, that examined effects of additions of organic and inorganic Se mixture, reported that the sources and levels of Se did not affect the litter performance [11,12].

As with the results of previous studies, the mixed addition of Se to the lactation sow diet during the 4-week lactation period did not negatively affect the litter performance of the lactating sows. However, when organic and inorganic Se were mixed, there were few reports of the reproductive performance of lactating sows and the growth performance of their progeny. In addition, most of the experimental periods were during the whole gestation or late gestation, and there were few experiments during the lactation period [22]. In this experiment, there was no significant difference in the growth performance of piglets, despite the addition of organic and inorganic Se to the diet. This result is considered to be because the experimental period was set to the lactation period only.

### Milk composition

The effects of dietary selenium in the lactation diet on the milk composition of sows during lactation are shown in (Table 4). The fat, protein, lactose, total solid and solid nonfat content in both colostrum and milk were not affected by mixed addition levels of Se during lactation.

Newborn piglets receive Se from sow's milk and colostrum, and the sources and levels of Se in the sow's diet influence the Se status of nursing and weaning piglets [23]. The Se concentration in mammary secretions can be increased by dietary supplementation with minerals, and the organic form of Se used in dietary supplements is more effective at increasing the Se concentration in colostrum and milk than the inorganic form [24,25].

Previous studies have shown that the Se concentration in sows fed a Se mixture (0.15 ppm organic + 0.15 ppm inorganic) were similar to those treated with 0.15 ppm organic Se. In addition, as the amount of Se added increased, the Se concentration of sow milk increased [11,12]. In the present study, there was no significant difference in the Se concentration in milk, despite the addition of organic and inorganic Se to the diet. This is considered to be because the experimental period was set as the lactation period, as above.

### Serum Se concentration in lactating sows and piglets

The effects of dietary selenium on serum the selenium concentration in sows and piglets are shown in Table 5). The serum Se concentrations of sows and piglets at 7 days of lactation were significantly higher when sows were fed organic and inorganic Se than when they were fed the control Se (p<0.01; respectively). However, there was no significant difference between the ISOS treatment groups.

Selenium can be passed on to offspring in the placenta and pig milk [26]. In addition, higher Se concentrations in sow milk increased Se status in weaning pigs [27]. Organic Se is more effective than inorganic Se in increasing the Se content in the blood, and persists longer in the body of animals [28].

Previous studies have shown that serum Se concentrations in sows and piglets fed a Se mixture (0.15 ppm organic + 0.15 ppm inorganic) were similar to those treated with 0.15 ppm organic Se [12], and the Se concentration in the serum increased as the level of Se addition increased [11].

In this study, ISOS15 and ISOS25 treatments resulted in significantly higher in serum Se concentrations in sows and piglets than in the control group, and there was no significant difference between the ISOS treatment groups. This result means that the Se concentration in piglet serum was increased by ingesting colostrum and milk secreted by lactating sows, regardless of the level of mixed addition of organic and inorganic Se to the lactating sow diet.

### Tissue Se concentration in piglets

The effects of dietary selenium in the lactation diet on tissue selenium concentration in piglets are shown in (Table 6). The kidney and muscle Se concentrations of piglets at 21 days of lactation were significantly higher when sows were fed organic and inorganic Se than the control (p = 0.03; p = 0.04; respectively). However, there was no significant difference between the ISOS treatment groups.

In general, Se concentrations in porcine tissues vary significantly from tissue to tissue [29]; in previous studies, it was reported that selenium's ability to accumulate in tissues gradually decreases in the order of kidney, liver, and muscle [30]. Mahan and Peters [12] reported that Se supplementation in a sow diet during pregnancy or lactation increased the Se content in sow and piglet tissues.

In the present study, ISOS15 and ISOS25 treatments resulted in significantly higher tissue Se concentrations in piglets than the control group, and there was no significant difference between the ISOS treatment groups. This result demonstrates that the Se concentration in piglet tissue was increased by ingesting colostrum and milk secreted by lactating sows, regardless of the level of mixed addition of organic and inorganic Se to the lactating sows diet.

## CONCLUSION

Supplementation with mixed inorganic and organic selenium in a lactating diet could improve the selenium status of sows and piglets.

## Figures and Tables

**Table 1:** The chemical composition of the experimental diet for lactating sow

Item	Treatment		

	Control	ISOS15^1)^	ISOS25^2)^
Ingredient (%)			
Corn	75.48	75.48	75.48
SBM-46	15.53	15.53	15.53
Wheat bran	3.00	3.00	3.00
Tallow	1.72	1.72	1.72
Limestone	1.28	1.28	1.28
MCP	1.49	1.49	1.49
L-methionine (90%)	0.04	0.04	0.04
L-lysine sulfate (50%)	0.59	0.59	0.59
Threonine (98.5%)	0.17	0.17	0.17
Vitamin premix^3)^	0.10	0.10	0.10
Mineral premix^4)^	0.10	0.10	0.10
Choline chloride-50	0.10	0.10	0.10
Salt	0.40	0.40	0.40
Sum	100.00	100.00	100.00
Chemical composition^5)^			
ME (kcal/kg)	3,300.00	3,300.00	3,300.00
CP (%)	13.49	13.49	13.49
Lysine (%)	0.96	0.96	0.96
Methionine (%)	0.26	0.26	0.26
Threonine (%)	0.65	0.65	0.65
Ca (%)	0.76	0.76	0.76
Total P (%)	0.65	0.65	0.65

SBM, soybean meal; MCP, monocalcium phosphate; ME, metabolizable energy; CP, crude protein.

1) ISOS15: Basal diet + inorganic selenium 0.15 ppm + organic selenium 0.15 ppm.

2) ISOS25: Basal diet + inorganic selenium 0.25 ppm + organic selenium 0.25 ppm.

3) Provided the following per kilogram of diet: vitamin A, 12,000 IU; vitamin D_3_, 1,200 IU; vitamin E, 68 IU; vitamin K, 5.0 mg; thiamine (vitamin B_1_), 2.60 mg; riboflavin (vitamin B_2_), 7.8 mg; niacin (vitamin B_3_), 60 mg; pyridoxine (vitamin B_6_), 6.00 mg; d-biotin, 0.5 mg; folic acid, 6.0 mg; vitamin B_12_, 0.02 mg.

4) Provided the following per kilogram of diet, control diet: Se, 0 mg; I, 0.75 mg; Mn, 60 mg; Cu, 60 mg; Fe, 120 mg; Zn, 46 mg; Co 0.4 mg; ISOS15: inorganic Se, 0.15 ppm; organic Se. 0.15 ppm; I, 0.75 mg; Mn, 60 mg; Cu, 60 mg; Fe, 120 mg; Zn, 46 mg; Co, 0.4 mg; ISOS25: inorganic Se, 0.25 ppm; organic Se, 0.25 ppm; I, 0.75 mg; Mn, 60 mg; Cu, 60 mg; Fe, 120 mg; Zn, 46 mg; Co, 0.4 mg.

5) Calculated values.

**Table 2:** Effects of supplemental selenium in the lactation diet on the body weight and backfat thickness of sows during lactation

Criteria	Treatment^1)^			SEM	p-value

	Control	ISOS15	ISOS25		
Body weight (kg)					
24 h postpartum	239.85	246.00	239.78	5.266	0.86
7 d of lactation	248.10	254.76	248.98	5.265	0.82
21 d of lactation	234.33	240.86	235.00	5.668	0.79
Changes (21–0 d)	−5.51	−5.13	−4.78	2.496	0.99
Backfat thickness (mm)					
24 h postpartum	19.08	20.58	17.25	1.051	0.39
7 d of lactation	18.41	20.25	17.66	0.983	0.55
21 d of lactation	17.41	18.33	17.41	0.910	0.89
Changes (21–0 d)	−1.66	−2.25	0.16	0.689	0.30
Average daily feed intake (kg)	5.40	5.87	6.00	0.212	0.41

SEM, standard error of means.

1) Treatment: Control: corn–soybean meal (SBM) based diet, ISOS15: corn–soybean meal based diet with inorganic selenium 0.15 ppm + organic selenium 0.15 ppm, ISOS25: corn–soybean meal based diet with inorganic selenium 0.25 ppm + organic selenium 0.25 ppm.

**Table 3:** Effects of supplemental selenium in the lactation diet on the performance of sows during lactation

Criteria	Treatment^1)^			SEM	p-value

	Control	ISOS15	ISOS25		
Number of piglets					
After cross-fostering^2)^	------------------------------------- 12.00 ------------------------------------			5.266	0.86
7 d of lactation	11.50	11.66	11.50	0.120	0.75
21 d of lactation	11.16	11.16	11.33	0.206	0.94
Litter weight (kg)					
After cross-fostering	16.93	15.88	15.69	0.700	0.29
7 d of lactation	29.63	29.81	28.97	1.128	0.95
21 d of lactation	63.73	59.30	61.90	1.857	0.66
Litter weight gain	46.79	40.72	46.20	1.675	0.32
Piglet weight (kg)					
After cross-fostering	1.40	1.54	1.30	0.058	0.29
7 d of lactation	2.58	2.55	2.51	0.091	0.96
21 d of lactation	5.74	5.38	5.47	0.168	0.70
Piglet weight gain	4.33	3.84	4.16	0.150	0.41

SEM, standard error of means.

1) Treatment: Control: corn–soybean meal (SBM) based diet, ISOS15: corn–soybean meal based diet with inorganic selenium 0.15 ppm + organic selenium 0.15 ppm, ISOS25: corn–soybean meal based diet with inorganic selenium 0.25 ppm + organic selenium 0.25 ppm.

2) After cross-fostering day within 24 h postpartum.

**Table 4:** Effects of supplemental selenium in the lactation diet on the milk composition of sows during lactation

Criteria	Treatment^1)^			SEM	p-value

	Control	ISOS15	ISOS25		
Fat (%)					
Colostrum	------------------ 8.14 -----------------			-	-
7 d of lactation	6.82	6.91	6.77	0.028	0.52
21 d of lactation	6.75	6.83	6.75	0.026	0.66
Protein (%)					
Colostrum	------------------ 11.86 -----------------			-	-
7 d of lactation	4.42	4.57	4.46	0.178	0.51
21 d of lactation	4.38	4.44	4.47	0.459	0.27
Lactose (%)					
Colostrum	------------------ 3.98 ------------------			-	-
7 d of lactation	5.98	5.68	5.75	0.032	0.48
21 d of lactation	5.94	5.75	5.63	0.132	0.26
Total solid (%)					
Colostrum	------------------ 26.50 --------------			-	-
7 d of lactation	17.25	17.38	17.19	0.258	0.45
21 d of lactation	17.65	17.53	17.43	0.543	0.58
Solid not fat (%)					
Colostrum	------------------ 15.46 ----------------			-	-
7 d of lactation	10.55	10.68	10.64	0.058	0.58
21 d of lactation	10.49	10.54	10.61	0.081	0.25
Selenium (ppm)					
Colostrum	------------------ 0.10 ------------------			-	-
7 d of lactation	0.07	0.07	0.08	0.003	0.58
21 d of lactation	0.07	0.07	0.08	0.005	0.50

SEM, standard error of means.

1) Treatment: Control: corn–soybean meal (SBM) based diet, ISOS15: corn–soybean meal based diet with inorganic selenium 0.15 ppm + organic selenium 0.15 ppm, ISOS25: corn–soybean meal based diet with inorganic selenium 0.25 ppm + organic selenium 0.25 ppm.

**Table 5:** Effects of supplemental selenium on the serum selenium concentration in sows and piglets

Criteria	Treatment^1)^			SEM	p-value

	Control	ISOS15	ISOS25		
Sow					
Selenium (ppm)					
24 h postpartum	----------------------------------------- 0.13 -----------------------------------------			-	-
7 d of lactation	0.11^a^	0.26^b^	0.21^b^	0.021	<0.01
21 d of lactation	0.24	0.29	0.27	0.013	0.52
Piglet					
Selenium (ppm)					
24 h postpartum	----------------------------------------- 0.03 -----------------------------------------			-	-
7 d of lactation	0.06^a^	0.11^b^	0.19^b^	0.008	<0.01
21 d of lactation	0.13	0.14	0.18	0.011	0.08

SEM, standard error of means.

1) Treatment: Control: corn–soybean meal (SBM) based diet, ISOS15: corn–soybean meal based diet with inorganic selenium 0.15 ppm + organic selenium 0.15 ppm, ISOS25: corn–soybean meal based diet with inorganic selenium 0.25 ppm + organic selenium 0.25 ppm.

a,b Means with different superscripts in the same row significantly differ (p<0.05).

**Table 6:** Effects of supplemental selenium in the lactation diet on the tissue selenium concentration in piglets

Criteria	Treatment^1)^			SEM	p-value

	Control	ISOS15	ISOS25		
Selenium (ppm)					
Liver					
24 h postpartum	--------------------------------- 0.13 ---------------------------------			-	-
21 d of lactation	0.41	0.46	0.53	0.063	0.84
Kidney					
24 h postpartum	--------------------------------- 0.03 ---------------------------------			-	-
21 d of lactation	0.34^a^	0.38^b^	0.42^b^	0.156	0.03
Muscle					
24 h postpartum	--------------------------------- 0.03 ---------------------------------			-	-
21 d of lactation	0.13^a^	0.34^b^	0.43^b^	0.120	0.04

SEM, standard error of means.

1) Treatment: Control: corn–soybean meal (SBM) based diet, ISOS15: corn–soybean meal based diet with inorganic selenium 0.15 ppm + organic selenium 0.15 ppm, ISOS25: corn–soybean meal based diet with inorganic selenium 0.25 ppm + organic selenium 0.25 ppm.

a,b Means with different superscripts in the same row significantly differ (p<0.05).
